# Supplementary material for: Effects of Helicobacter pylori treatment on the incidences of autoimmune diseases and inflammatory bowel disease in patients with diabetes mellitus
Source: PLoS One. 2022 May 23;17(5):e0265323. doi: 10.1371/journal.pone.0265323 (PMC9126384; doi:10.1371/journal.pone.0265323)
Supplement: S1 Table — (DOC) [file pone.0265323.s001.doc]

**S1 Table.**

| **Demographic prevalence of PUD+HPRx and PUD-HPRx in a DM population and a general population post-propensity score-matching.** | | | | | |
| --- | --- | --- | --- | --- | --- |
|  | PUD+HPRx in GP | PUD-HPRx in GP | PUD+HPRx in DM | PUD-HPRx in DM |  |
| N | 13,713 | 48,541 | 48,737 | 48,737 | p-value |
| Age in years (Mean±SD)* | 50.82 (±15.19) | 58.30 (±14.50) | 58.11 (±14.12) | 58.23 (±14.24) | 0.129 |
| Age categories (N, %) |  |  |  |  |  |
| <35 | 2,047 (14.96%) | 2,462 (5.07%) | 2,511 (5.15%) | 2,510 (5.15%) | 0.0613 |
| 35-44 | 3,062 (22.32%) | 5,682 (11.71%) | 5,817 (11.94%) | 5,815 (11.93%) |  |
| 45-54 | 3,458 (25.19%) | 11,599 (23.90%) | 11,800 (24.21%) | 11,802 (24.22%) |  |
| 55-64 | 2,317 (16.91%) | 11,652 (24.00%) | 11,898 (24.41%) | 11,898 (24.41%) |  |
| 65+ | 2,829 (20.63%) | 17,146 (35.32%) | 16,711 (34.29%) | 16,712 (34.29%) |  |
|  |  |  |  |  |  |
| Gender (N, %)* |  |  |  |  |  |
| Female | 8,222 (59.96%) | 26,509 (54.61%) | 26,857 (55.11%) | 26,854 (55.10%) | 0.2059 |
| Male | 5,491 (40.04%) | 22,032 (45.39%) | 21,880 (44.89%) | 21,883 (44.90%) |  |
| Income status (N, %)* |  |  |  |  |  |
| Dependent | 2,799 (20.41%) | 12,278 (25.29%) | 12,201 (25.03%) | 12,199 (25.03%) | 0.2106 |
| <20,000 | 3,470 (25.30%) | 12,799 (26.37%) | 12,645 (25.95%) | 12,647 (25.95%) |  |
| 20,000-40,000 | 4,896 (35.70%) | 16,920 (34.86%) | 17,099 (35.08%) | 17,100 (35.09%) |  |
| ≥ 40,000 | 2,548 (18.58%) | 6,544 (13.48%) | 6,792 (13.94%) | 6,791 (13.93%) |  |
| Comorbidities (N, %) |  |  |  |  |  |
| Hypertension (No) | 10,184 (74.27%) | 26,550 (54.70%) | 26,678 (54.74%) | 26,684 (54.75%) |  |
| Hypertension * | 3,529 (25.73%) | 21,991 (45.30%) | 22,059 (45.26%) | 22,053 (45.25%) | 0.9838 |
| Hyperlipidemia (No) | 10,805 (78.79%) | 29,560 (60.90%) | 29,509 (60.55%) | 29,509 (60.55%) |  |
| Hyperlipidemia* | 2,908 (21.21%) | 18,981 (39.10%) | 19,228 (39.45%) | 19,228 (39.45%) | 0.4362 |
| Myocardial infraction (No) | 13,603 (99.20%) | 47,615 (98.09%) | 47,922 (98.33%) | 47,922 (98.33%) |  |
| Myocardial infraction | 110 (0.80%) | 926 (1.91%) | 815 (1.67%) | 815 (1.67%) | 0.0054 |
| Congestive heart failure (No) | 13,403 (97.74%) | 46,315 (95.41%) | 46,455 (95.32%) | 46,479 (95.37%) |  |
| Congestive heart failure | 310 (2.26%) | 2,226 (4.59%) | 2,282 (4.68%) | 2,258 (4.63%) | 0.7741 |
| Peripheral vascular disease (No) | 13,568 (98.94%) | 47,674 (98.21%) | 47,955 (98.40%) | 47,959 (98.40%) |  |
| Peripheral vascular disease | 145 (1.06%) | 867 (1.79%) | 782 (1.60%) | 778 (1.60%) | 0.0326 |
| Cerebral vascular disease (No) | 13,003 (94.82%) | 42,849 (88.27%) | 44,096 (90.48%) | 43,437 (89.13%) |  |
| Cerebral vascular disease | 710 (5.18%) | 5,692 (11.73%) | 4,641 (9.52%) | 5,300 (10.87%) | <.0001 |
| Dementia (No) | 13,607 (99.23%) | 47,549 (97.96%) | 47,931 (98.35%) | 47,818 (98.11%) |  |
| Dementia | 106 (0.77%) | 992 (2.04%) | 806 (1.65%) | 919 (1.89%) | <.0001 |
| Chronic kideney disease (No) | 13,300 (96.99%) | 45,521 (93.78%) | 46,189 (94.77%) | 46,104 (94.60%) |  |
| Chronic kideney disease | 413 (3.01%) | 3,020 (6.22%) | 2,548 (5.23%) | 2,633 (5.40%) | <.0001 |
| Cancer (No) | 13,087 (95.43%) | 45,313 (93.35%) | 45,489 (93.34%) | 45,499 (93.36%) |  |
| Cancer* | 626 (4.57%) | 3,228 (6.65%) | 3,248 (6.66%) | 3,238 (6.64%) | 0.9914 |
| Charlson's Index Score (Mean±SD) | 1.39 (±1.44) | 1.99 (±1.75) | 1.99 (±1.71) | 2.01 (±1.75) | 0.4738 |
| Charlson's Index Categories (N, %)* |  |  |  |  |  |
| 0 | 3,328 (24.27%) | 6,868 (14.15%) | 6,854 (14.06%) | 6,856 (14.07%) | 0.8841 |
| 1-2 | 5,941 (43.32%) | 16,036 (33.04%) | 15,973 (32.77%) | 15,974 (32.78%) |  |
| 3 | 2,474 (18.04%) | 12,116 (24.96%) | 12,157 (24.94%) | 12,155 (24.94%) |  |
| >=4 | 1,970 (14.37%) | 13,521 (27.85%) | 13,753 (28.22%) | 13,752 (28.22%) |  |
| Medication (N, %) |  |  |  |  |  |
| Metformin | 4,343(31.57%) | 18,461 (38.03%) | 43,147 (88.53%) | 41,014 (84.15%) | <.0001 |
| Sulfonylurea | 4,494(32.66%) | 19,101 (39.35%) | 43,940 (90.16%) | 41,846 (85.86%) | <.0001 |
| Dipeptidyl peptidase 4 inhibitor | 333(2.42%) | 1,850 (3.81%) | 5,704 (11.70%) | 4,913 (10.08%) | <.0001 |
| Insulin | 1,835(13.34%) | 9,500 (19.57%) | 20,856 (42.79%) | 19,617 (40.25%) | <.0001 |
| NSAIDs* | 11,313 (82.50%) | 41,493 (85.48%) | 41,749 (85.66%) | 41,751 (85.67%) | 0.642 |
| Antiplatelet agent | 1,157 (8.44%) | 6,394 (13.17%) | 7,207 (14.79%) | 6,277 (12.88%) | <.0001 |
| Warfarin | 60 (0.44%) | 488 (1.01%) | 439 (0.90%) | 467 (0.96%) | 0.2438 |
| Protom pump inhibitor | 10,344 (75.43%) | 22,189 (45.71%) | 40,232 (82.55%) | 26,823 (55.04%) | <.0001 |
| H2-receptor antagonist | 12,817 (93.47%) | 38,746 (79.82%) | 46,259 (94.92%) | 40,514 (83.13%) | <.0001 |
| Note: |  |  |  |  |  |
| * : variables used in the propensity score matching model.  GP, general population; H2-receptor antagonist, Histamine-2-receptor antagonist | |  |  |  |  |
