# Supplementary material for: Effects of Helicobacter pylori treatment on the incidences of autoimmune diseases and inflammatory bowel disease in patients with diabetes mellitus
Source: PLoS One. 2022 May 23;17(5):e0265323. doi: 10.1371/journal.pone.0265323 (PMC9126384; doi:10.1371/journal.pone.0265323)
Supplement: S2 Table — (DOC) [file pone.0265323.s002.doc]

**S2 Table.**

| **Autoimmune disease and inflammatory bowel disease outcomes in the matched cohorts.** | | | | | |
| --- | --- | --- | --- | --- | --- |
|  | PUD+HPRx | PUD-HPRx in GP | PUD+HPRx in DM | PUD-HPRx in DM | P-value |
| N | 13,713 | 48,541 | 48,737 | 48,737 |  |
| Two-year wash out period |  |  |  |  |  |
| Total follow-up person years  (in years) | 101,868.20 | 324,794.24 | 344,539.06 | 334,239.47 |  |
| Health outcomes (N,%) |  |  |  |  |  |
| Autoimmune disease | 634 (4.62%) | 1,511 (3.11%) | 2,253 (4.62%) | 1,560 (3.20%) | <.0001 |
| IBD | 609 (4.44%) | 1,302 (2.68%) | 2,349 (4.82%) | 1,338 (2.75%) | <.0001 |
| IBD and Asacol/Azathioprine | 16 (0.12%) | 21 (0.04%) | 58 (0.12%) | 24 (0.05%) | <.0001 |
| Incidence rate ratio (95%CI) |  |  |  |  |  |
| IBD (ref.=PUD-HPRx in GP) | 1.491 (1.041,1.942)** | ref. | 1.701 (1.589,1.821)*** | 0.999 (0.925,1.079) |  |
| IBD (ref.=PUD-HPRx in DM) | 1.493(0.986,2.001) | 1.001 (0.927,1.082) | 1.703 (1.592,1.823)*** | ref. |  |
| All-cause mortality |  |  |  |  |  |
| Total follow-up person years | 104,353.89 | 329,944.27 | 354,406.36 | 339,675.73 |  |
| All-cause mortality | 1,321 (9.63%) | 8,348 (17.20%) | 9,737 (19.98%) | 9,958 (20.43%) |  |
|  |  |  |  |  |  |
| Mortality rate ratio (95%CI) |  |  |  |  |  |
| Mortality (ref.=PUD-HPRx in GP) | 0.501 (0.317,0.684)** | ref. | 1.086 (1.054,1.118)*** | 1.159(1.125,1.193)*** |  |
| Mortality (ref.=PUD-HPRx in DM) | 0.432(0.384,0.479)*** | 0.863(0.838,0.889)*** | 0.937 (0.911,0.964)*** | ref. |  |
| Note: |  |  |  |  |  |
| *: p<0.05 **: p<0.01 *** p<0.001  GP, general population; DM, diabetes mellitus; IBD, inflammatory bowel disease. | | | | | |
