# Supplementary material for: Effects of Helicobacter pylori treatment on the incidences of autoimmune diseases and inflammatory bowel disease in patients with diabetes mellitus
Source: PLoS One. 2022 May 23;17(5):e0265323. doi: 10.1371/journal.pone.0265323 (PMC9126384; doi:10.1371/journal.pone.0265323)
Supplement: S3 Table — (DOC) [file pone.0265323.s003.doc]

**S3 Table.**

| **Cox proportion model results for Outcomes of IBD and mortality among matched cohorts** | | |
| --- | --- | --- |
|  | **IBD incidence with one year washout period** | **IBD incidence with two year washout period** |
| Variables | HR (95%CI) | HR (95%CI) |
| Medication (N, %) |  |  |
| Metformin (ref.: No) |  |  |
| PUD+HPRx in DM | 1.088 (0.960,1.233) | 1.098 (0.959,1.258) |
| PUD-HPRx in DM | 0.978 (0.852,1.121) | 0.974 (0.839,1.130) |
| Sulfonylurea (ref.: No) |  |  |
| PUD+HPRx in DM | 1.132 (0.990,1.295) | 1.084 (0.940,1.250) |
| PUD-HPRx in DM | 1.080 (0.932,1.251) | 1.113 (0.946,1.309) |
| Dipeptidyl peptidase 4 inhibitor (ref.: No) |  |  |
| PUD+HPRx in DM | 0.986 (0.878,1.107) | 0.960 (0.846,1.088) |
| PUD-HPRx in DM | 1.069 (0.917,1.247) | 1.085 (0.920,1.280) |
| Insulin (ref.: No) |  |  |
| PUD+HPRx in DM | 1.008 (0.934,1.089) | 1.022 (0.940,1.110) |
| PUD-HPRx in DM | 1.056 (0.956,1.167) | 1.014 (0.909,1.131) |
| *: p<0.05 **: p<0.01 *** p<0.001 |  |  |
