# Supplementary material for: Effects of Helicobacter pylori treatment on the incidences of autoimmune diseases and inflammatory bowel disease in patients with diabetes mellitus
Source: PLoS One. 2022 May 23;17(5):e0265323. doi: 10.1371/journal.pone.0265323 (PMC9126384; doi:10.1371/journal.pone.0265323)
Supplement: Attachment — Submitted filename: Response to Reviewers.doc [file pone.0265323.s004.doc]

Prof. Yoshio Yamaoka

Academic Editor, PLOS ONE

Dear Professor Yoshio Yamaoka:

I sincerely thank you for your processing our manuscript “PONE-D-21-21394
Effects of Helicobacter pylori eradication on the incidences of autoimmune diseases and inflammatory bowel disease in patients with diabetes mellitus” and for your decision. We also would like to express our cordial appreciation to reviewers for their excellent suggestions. As reviewer’s suggestion, we created supporting tables. Additionally, we have carefully revised our manuscript according to your comments. Our point-by-point responses are listed below.

**Editor’s comment:**

Comment 1:

Please ensure that your manuscript meets PLOS ONE's style requirements, including those for file naming.

Answer:

We had modified our manuscript to meet the PLOS ONE's style requirements, and renamed our figures according to the cited order in manuscript. Thanks for your kind reminder.

Comment 2:

This is a retrospective study with no control group. As such, we do not feel that any conclusions on the intervention effects can be supported; as such, we ask that you revise the text (especially, but no limited to, the aims and Conclusions) to avoid unsupported statements.

Answer:

Our control groups were PUD-HPRx in DM and PUD-HPRx in GP group. We compared the incidence of IBD and mortality of PUD+HPRx in DM group to other two groups. Besides, we presented the result included PUD+HPRx in GP group in the supporting tables.

Comment 3:

Thank you for stating the following in the Acknowledgments Section of your manuscript:“This study was funded by a grant from the Ministry of Health and Welfare (MOHW108-TDU-B-212-133006), Kaohsiung Medical University (109CM-KMU- 012), Kaohsiung Medical University Hospital (SA10905), and Kaohsiung Municipal Ta-Tung Hospital (KMTTH-107-014), which had no role in the study design, data analysis, data interpretation, or writing of the manuscript.”

We note that you have provided additional information within the Acknowledgements Section that is not currently declared in your Funding Statement. Please note that funding information should not appear in the Acknowledgments section or other areas of your manuscript. We will only publish funding information present in the Funding Statement section of the online submission form.

Please remove any funding-related text from the manuscript and let us know how you would like to update your Funding Statement. Currently, your Funding Statement reads as follows:“The author(s) received no specific funding for this work.”

Please include your amended statements within your cover letter; we will change the online submission form on your behalf.

Answer:

Thank you for your suggestion. We would remove the funding-related text from the manuscript. Please change our Funding Statement to

“This study was funded by a grant from the Ministry of Health and Welfare (MOHW108-TDU-B-212-133006), Kaohsiung Medical University (109CM-KMU- 012), Kaohsiung Medical University Hospital (SA10905), and Kaohsiung Municipal Ta-Tung Hospital (KMTTH-107-014), which had no role in the study design, data analysis, data interpretation, or writing of the manuscript. ”

**Reviewer 1:**

Comment 1:

Due to the retrospective observation study design, the findings are vulnerable to many biases. Why DM patients were selected as the main study group? Because one of control group was PUD patients not treated for H. pylori in general population (PUD-HpRx in GP), PUD patients who were treated for Hp infection in general population might be better to assess the Hp treatment and AD or IBD association.

Answer:

We selected DM patients as the main study group because DM and PUD are common disease in Taiwan. The data of DM patients were more intact in our database. The supporting table include PUD patients who were treated for Hp infection in general population (PUD+HpRx in GP). The result was similar. The sample of PUD+HpRx in GP group was too small to match to other groups.

Comment 2:

PUD is a well-known indication for H. pylori treatment since 1990s. Why so many PUD patients in this database had not treated for H. pylori? Especially in DM group, the number who did not receive H. pylori treatment seems more than 10 times in number. This might raise a question about the completeness or correctness of the database.

Answer:

In Taiwan, H. pylori biopsy will be performed during Esophagogastroduodenoscopy (EGD). If H. pylori infection is confirmed, H. pylori eradication will be given. But the report of H. pylori biopsy do not present in the database. This limitation makes us unable to exclude the rare patients with H. pylori infection but refused or unable to receive H. pylori treatment. But it doesn’t influence our result. Due to the patients with H. pylori infected almost always receive treatment in Taiwan, the PUD patients not received H. pylori treatment may represent negative results of H. pylori infection.

Comment 3:

The persons in the control groups who were not treated for H. pylori in general population or in DM patients should be very heterogenous. The persons are one of the cases; 1) H. pylori uninfected persons, 2) H. pylori infected but not treated.

Answer:

Yes. The condition(H. pylori infected but not treated) is rare in Taiwan. Due to the patients with H. pylori infected almost always receive treatment in Taiwan, the PUD patients not received H. pylori treatment may represent negative results of H. pylori infection.

Comment 4:

The persons in the case group who were treated for H. pylori also seems very heterogenous. Those are one of the cases; 1) H. pylori treatment medication was prescribed but not took at all (poor compliance), 2) H. pylori treatment medication was took, but H. pylori eradication was either successful or failed. Since the eradication success data are not available from the database, the title and description needs be modified to more modest term such as H. pylori treatment not H. pylori eradication. Actually it’s very hard to tell whether exposure to H. pylori treatment as whole, H. pylori eradication itself, or just an exposure of certain antibiotic included in the H. pylori treatment is the main culprit for the authors finding.

Answer:

Yes. The result of H. pylori treatment can’t be presented in the database. It’s one of the limitation in our study. The success rate of H. pylori treatment in Taiwan was 82-94% under 7 days triple therapy, and 84% under third-line therapy. We had modified at Discussion section, paragraph 7, line 12~13. We will modify the term H. pylori eradication to H. pylori treatment. Thanks for your suggestion.

We define the patients with H. pylori treatment by the set of triple or quadruple therapy for more than 7 days as our previous study by Lin et al. (Clin Gastroenterol Hepatol. 2019 Sep;17(10):1991-1999) The therapy include proton pump inhibitor twice daily use, which differ from antibiotic therapy for other infection.

Comment 5:

One of main finding was that H. pylori treatment decreased the all-cause mortality in the DM group with PUD. This finding might also be misleading because there is a chance that the patients having more severe DM were not treated for H. pylori infection. If mild DM patients were more selectively treated for H. pylori, the group should have better survival rates.

Answer:

The condition(H. pylori infected but not treated) is rare in Taiwan. The rare condition may happen in Intensive Care Unit when the patient is critical. However, our database was from Outpatient department where patients were stable. And the result had been adjusted by Charlson Comorbidity Index score.

Comment 6:

Please clarify the study entry date and end date. In line 140 on page 6, the persons who had received treatment for H. pylori infection between 1997 and 2013 were enrolled. In line 148 on page 6, the sentence was described as “The person-years of follow-up were estimated from the index date plus a two-year washout duration to the date of diagnosis of AD or IBD, or an end date of December 31, 2010.” The sentence might be interpreted as the end date of follow up of this study was on Dec 31, 2010, which is much earlier than 2013.

Answer:

The database is 1997-2013. We enrolled patients from 2000-2010 for more intact data. We had modified in line 136~137 on page 6.

Comment 7:

Discussion seems too short and more plausible link between IBD, AD and mortality needs be provided. Also, the limitations of this study should be described in detail.

Answer:

Thanks for your suggestion. We will revise the discussion section and limitations more completely.

**Reviewer 2:**

Comment 1:

Authors selected three groups. However, Authors should add one group: general population with peptic ulcer who received H. pylori treatment.

Answer:

The supporting table include PUD patients who were treated for Hp infection in general population (PUD+HpRx in GP). The result was similar. The sample of PUD+HpRx in GP group was too small to match to other groups.

Comment 2:

In this study, authors included patients with peptic ulcer. If authors enrolled patients with gastritis, not peptic ulcer, could the results be different?

Answer:

The coding of gastritis is not accurate because many patients with gastritis usually take oral medicine without received Esophagogastroduodenoscopy (EGD). However, patients with peptic ulcer were almost received EGD. So the coding of peptic ulcer is more accurate than gastritis.

Comment 3:

Although authors enrolled many patients, direct association with autoimmune diseases, inflammatory bowel disease and diabetes mellitus is unclear.

Answer:

Our results revealed that treatment of H. pylori infection was associated with the increased incidence rates of ADs and IBD in DM patients with PUD. The incidence rate of IBD in PUD-HPRx in DM group did not increase compared to PUD-HPRx in GP group. However, further studies should be performed.

Comment 4:

Why did authors select patients with diabetes mellitus?

Answer:

We selected DM patients as the main study group because DM and PUD are common disease in Taiwan. And the data of DM patients were more intact in our database. The supporting table include PUD patients who were treated for Hp infection in general population (PUD+HpRx in GP). The result was similar. The sample of PUD+HpRx in GP group was too small to match to other groups.

Comment 5:

H. pylori treatment decreased the all-cause mortality in the DM group with peptic ulcer. Why?

Answer:

H. pylori treatment also decreased the all-cause mortality in the GP group with peptic ulcer (see the supporting table). We speculated that treatment of H. pylori decreases the risks of gastric cancer and cardiovascular disease, which are increased in DM patients.

Comment 6:

What is the relationship between the time received H. pylori eradication therapy and the time of autoimmune diseases and IBD onset?

Answer:

The person-years of follow-up were estimated from the index date plus a two-year washout duration to the date of diagnosis of AD or IBD, or an end date. The H. pylori therapy may change intestinal microbiota, which may induce the incidence of AD or IBD.

Comment 7:

Congestive heart failure and use of anti-platelet agent are risk factor for IBD incidence with two-year washout period. Why?

Answer:

We speculate that using anti-platelet agent increase the risk of peptic ulcer, and more patients with peptic ulcer received EGD and H. pylori biopsy. Patients with congestive heart failure frequently use anti-platelet agent may lead to the same result. We added our speculation at Discussion section, paragraph 6. Thanks for your question.

Comment 8:

Authors should add data of intake, kinds and dose of medication for DM.

Answer:

The data of intake was not included in our database. We added the data of medications for DM in the table 1 and 3. Table 3 showed using metformin, sulfonylurea, and insulin increased the incidence of IBD, but DPP4 inhibitor did not. We performed the subgroup analysis to compare the incidence of IBD in patients with and without the specific DM medication in PUD+HPRx in DM and PUD-HPRx in DM group(see supporting table 2). The result showed DM medications didn’t increase the incidence of IBD. We added this finding at Discussion section, paragraph 7.

Comment 9:

Please add p values in Table 1.

Answer:

We will correct the table in the manuscript. Thanks for your kind reminder.

Comment 10:

What is a p value in Table 2? PUD+HPRx vs PUD-HPRx?

Answer:

We will correct the table in the manuscript. Thanks for your kind reminder.

Comment 11:

Several H. pylori bacterial virulence factors that dampen the immune response of the host were identified, including VacA, CagA, and H. pylori lipopolysaccharide. However, Western population infected with cagA-negative and vacA s2m2 type H. pylori. Therefore, influence of eradication therapy for IBD may differ between Asian population and Western population.

Answer:

Yes, the different types of H. pylori infection between Asian and Western population may influence the result of effect of H. pylori therapy for IBD. The types of H. pylori were not included in our database. It needs more study include Western population for provement.

Comment 12:

Authors used data received treatment for H. pylori infection between 1997 and 2013. How is the judgment for eradication therapy?

Answer:

The database is 1997-2013. We enrolled patients from 2000-2010 for more intact data. We had modified it in line 139~140 on page 6. Patients received H. pylori therapy before 2000 were grouped to PUD-HPRx. This doesn’t influence the result because these patients actually with higher risk of IBD were grouped to PUD-HPRx, which has lower risk of IBD.

Comment 13:

How about H. pylori infection rate in a group with peptic ulcer without H. pylori treatment?

Answer:

The condition (H. pylori infected but not treated) is rare in Taiwan. Due to the patients with H. pylori infection almost always receive treatment in Taiwan, the PUD patients not receive H. pylori treatment may represent negative results of H. pylori infection.

Kun-Der Lin, M.D., Ph. D.

Department of Internal Medicine, Kaohsiung Municipal Ta-Tung Hospital; Division of Endocrinology and Metabolism, Department of Internal Medicine, Kaohsiung Medical University Hospital, and College of Medicine, Kaohsiung Medical University, Kaohsiung, Taiwan

Phone: +886-7-2911101#8912

E-mail: berg.kmu@mail.com
