# Supplementary material for: Effects of Helicobacter pylori treatment on the incidences of autoimmune diseases and inflammatory bowel disease in patients with diabetes mellitus
Source: PLoS One. 2022 May 23;17(5):e0265323. doi: 10.1371/journal.pone.0265323 (PMC9126384; doi:10.1371/journal.pone.0265323)
Supplement: Attachment — Submitted filename: Response to Reviewers.docx [file pone.0265323.s005.docx]

Prof. Yoshio Yamaoka

Academic Editor, PLOS ONE

Dear Professor Yoshio Yamaoka:

I sincerely thank you for your processing our manuscript “PONE-D-21-21394

Effects of Helicobacter pylori eradication on the incidences of autoimmune diseases and inflammatory bowel disease in patients with diabetes mellitus” and for your decision. We also would like to express our cordial appreciation to reviewers for their excellent suggestions. As reviewer’s suggestion, we created supporting tables. Additionally, we have carefully revised our manuscript according to your comments. Our point-by-point responses are listed below.

**Reviewer 2:**

Comment 1:

Many abbreviations were used. Popular abbreviations will be acceptable, such as DM, IBD and PUD. However, minor abbreviations should be avoided to use, such as AD and GP.

Answer:

We had modified our manuscript to avoid use minor abbreviations such as AD and GP. Thanks for your kind reminder.

Comment 2:

IBD included Crohn’s disease and chronic ulcerative colitis. Pathogenesis of Crohn’s disease and chronic ulcerative colitis differ. Please show divide data into Crohn’s disease and chronic ulcerative colitis.

Answer:

We had divided the data of IBD to Crohn’s disease and chronic ulcerative colitis (see Table 2). Both the incidence of Crohn’s disease and chronic ulcerative colitis were significantly increased in PUD+HPRx in DM group as that of IBD. We had modified at Results section, paragraph 1, line 12~15 on page 8. Cox proportional hazards regression was performed on the outcomes of Crohn’s disease and chronic ulcerative colitis among the matched cohorts (Table S4) and the results were similar. Because the sample of chronic ulcerative colitis was too small that may produce bias in subgroup analysis, we just put the table below the response letter instead of manuscript.

Comment 3:

How about severity of IBD?

Answer:

The severity of IBD was defined by the clinical signs such as frequency of diarrhea, abdominal pain, fever, anemia…etc, which were not included in our database. We had modified at Discussion section, line 273~275 on page 12.

Comment 4:

Please add data of PPI/H2RA in Table.

Answer:

We had added data of PPI/H2RA in Table 1 and S1 table. The use of PPI significantly increased in PUD+HPRx in general population and DM groups due to H. pylori treatment. The use of H2RA significantly increased in PUD+HPRx in general population and DM groups, which may owing to more upper GI symptoms in patients with H. pylori infection.

Kun-Der Lin, M.D., Ph. D.

Department of Internal Medicine, Kaohsiung Municipal Ta-Tung Hospital; Division of Endocrinology and Metabolism, Department of Internal Medicine, Kaohsiung Medical University Hospital, and College of Medicine, Kaohsiung Medical University, Kaohsiung, Taiwan

Phone: +886-7-2911101#8912

E-mail: berg.kmu@mail.com

| **Table S4. Cox proportion model results for incidence of Crohn’s disease and Chronic ulcerative colitis with two year washout period among matched cohorts** | | |
| --- | --- | --- |
|  | Crohn’s disease incidence | Chronic ulcerative colitis incidence |
| Variables | HR (95%CI) | HR (95%CI) |
| Cohorts (ref. group: PUD-HPRx in DM) |  |  |
| PUD-HPRx in GP | 0.951 (0.875,1.033) | 0.938 (0.722,1.218) |
| PUD+HPRx in DM | 2.017 (1.877,2.167)*** | 1.842 (1.475,2.301)*** |
| Age categories (ref. group: <35) |  |  |
| 35-44 | 0.888 (0.790,0.999)*** | 0.991 (0.657,1.496) |
| 45-54 | 0.876 (0.785,0.978)*** | 0.961 (0.652,1.417) |
| 55-64 | 0.794 (0.708,0.891)*** | 0.906 (0.608,1.351) |
| 65+ | 0.798 (0.711,0.896)*** | 1.271 (0.859,1.881) |
| Gender (ref. female) |  |  |
| Male | 0.865 (0.816,0.916)*** | 0.938 (0.788,1.117) |
| Income status (ref.: dependent) |  |  |
| <20,000 | 1.110 (1.026,1.200)*** | 0.915 (0.721,1.162) |
| 20,000-40,000 | 1.056 (0.982,1.135) | 0.912 (0.732,1.136) |
| 40,000 | 0.920 (0.833,1.015) | 1.113 (0.833,1.486) |
| Comorbidities (N, %) |  |  |
| Hypertension (ref.: No) | 0.958 (0.900,1.019) | 0.958 (0.792,1.158) |
| Hyperlipidemia (ref.:No) | 0.832 (0.782,0.886)*** | 0.873 (0.723,1.054) |
| Myocardial infraction (ref.: No) | 0.946 (0.739,1.213) | 0.946 (0.482,1.857) |
| Congestive heart failure (ref.: No) | 1.084 (0.937,1.253) | 1.472 (1.021,2.121)** |
| Peripheral vascular disease (ref.:No) | 1.152 (0.934,1.421) | 0.837 (0.414,1.693) |
| Cerebral vascular disease (ref.:No) | 0.961 (0.863,1.071) | 1.031 (0.764,1.392) |
| Dementia (ref.:No) | 0.959 (0.739,1.245) | 0.649 (0.286,1.473) |
| Chronic kideney disease (ref.:No) | 0.997 (0.867,1.145) | 0.909 (0.603,1.369) |
| Cancer (ref.:No) | 0.981 (0.858,1.121) | 0.889 (0.596,1.326) |
| Charlson's Index Categories (ref.: CCI=0) |  |  |
| 1-2 | 1.066 (0.982,1.156) | 1.246 (0.944,1.643) |
| 3 | 1.100 (1.006,1.202)* | 1.384 (1.030,1.860)** |
| >=4 | 1.075 (0.969,1.192) | 1.301 (0.926,1.829) |
| Medication (N, %) |  |  |
| Metformin (ref.: No) | 1.132 (1.011,1.295)** | 1.109 (1.008,1.216)** |
| Sulfonylurea (ref.: No) | 1.109 (0.868,1.416) | 1.160 (0.932,1.443) |
| Dipeptidyl peptidase 4 inhibitor (ref.: No) | 1.025 (0.925,1.136) | 1.056 (0.956,1.167) |
| Insulin (ref.: No) | 1.084 (0.972,1.209) | 1.107 (1.041,1.178)** |
| NSAIDs (ref.: No) | 1.467 (1.338,1.608)*** | 1.319 (1.009,1.725)* |
| Antiplatelet agent (ref.: No) | 1.185 (1.090,1.287)*** | 1.298 (1.021,1.649)* |
| Warfarin (ref.: No) | 0.746 (0.520,1.071) | 0.877 (0.725,1.003) |
| Note: |  |  |
| *: p<0.05 **: p<0.01 *** p<0.001 |  |  |
